# Knowledge and attitudes about influenza vaccination in rheumatic diseases patients

**DOI:** 10.1080/21645515.2020.1816108

**Published:** 2020-09-29

**Authors:** Gabriel Figueroa-Parra, Jorge Antonio Esquivel-Valerio, Leticia Santoyo-Fexas, Andrea Moreno-Salinas, Carmen Magdalena Gamboa-Alonso, Ana Laura De Leon-Ibarra, Dionicio Angel Galarza-Delgado

**Affiliations:** Rheumatology Service, University Hospital “Dr. José Eleuterio González”, Universidad Autónoma de Nuevo León, Monterrey, México

**Keywords:** Influenza, vaccination, rheumatic diseases, rheumatoid arthritis, public health, vaccine hesitancy, vaccine acceptance

## Abstract

Patients with rheumatic diseases (RD) have a higher risk of morbidity and mortality from vaccine-preventable infections attributed to disease activity, comorbidities, immunosuppressive therapy, and other factors. Vaccines are one of the safest and most effective public health interventions. The aim of this study was to investigate knowledge and attitudes about influenza vaccination as factors influencing vaccine uptake and hesitancy in a population with RD. A descriptive cross-sectional study was designed. A self-administered questionnaire surveyed age, RD diagnosis, ten questions about the uptake, safety and efficacy of influenza vaccine, knowledge of cost-free availability, and the relationship between influenza vaccination and RD. A total of 223 questionnaires were filled; 79.8% of patients were vaccinated for influenza at least once. Uptake by diagnosis was 80.3% in rheumatoid arthritis, 76.2% in osteoarthritis, 86.7% in lupus, 73.9% in other auto-immune diseases (AID), and 60% in other non-AID; 83.9% of patients considered influenza vaccine as safe and effective. From those who had never been vaccinated, 26.7% of patients did not consider influenza vaccine safe and effective vs. 13.5% among patients who had been vaccinated (*P* = .032). Only 7.6% considered that RD patients could not be vaccinated; 11.7% thought that influenza vaccine would worsen their RD symptoms. This study showed that concerns about safety, efficacy, side effects, fear of the vaccine, and knowledge of cost diminished vaccine uptake. These are factors related to confidence, complacency, and convenience as components of vaccine hesitancy that affect influenza vaccination in RD patients.

## Introduction

Patients with rheumatic diseases (RD) have a higher risk of morbidity and mortality from vaccine-preventable infections attributed to disease activity, comorbidities, immunosuppressive therapy, and other factors.^1-4^ In the general population, approximately 1 in 10 unvaccinated adults were estimated to be annually infected by influenza, being symptomatic in half of these estimates.^5^ A recent systematic review reported an increased incidence of influenza and other vaccine-preventable infections in patients with RD.^4^ Rheumatoid arthritis (RA) is associated with an increased risk of influenza and its complications (pneumonia, stroke, and myocardial infarction).^2^ Other RD (particularly psoriatic arthritis, ankylosing spondylitis, and spondyloarthropathy)^3^ treated with biologic disease-modifying anti-rheumatic drugs (DMARD) have been associated with a higher incidence of influenza-like illness compared with the general population.^6^ In a cohort of ANCA-associated vasculitis, the combined incidence of influenza and pneumonia was higher compared to the general population.^7^

Vaccines are one of the safest and most effective public health interventions.^8^ Influenza vaccination had shown to reduce the incidence, complications, admissions, and mortality from influenza in patients with RD.^9^ In their 2019 recommendations for vaccinations in adult patients with autoimmune inflammatory rheumatic diseases, the European League Against Rheumatism established that influenza vaccination should be strongly considered for most patients with RD.

Vaccine hesitancy is one of the ten threats to global health established by the World Health Organization (WHO),^10^ and it is influenced by three main factors: 1) confidence, which is the lack of trust in the vaccine or provider; 2) complacency, which is the perception that there is no value or a need for a vaccine; and 3) convenience, which refers to the perceived lack of access or services toward vaccination. Influenced by these factors, vaccine-hesitant individuals may refuse some vaccines or delay vaccination, some individuals may refuse vaccines altogether.^11^ Among RD patients who had never been vaccinated, there are more concerns about safety and efficacy that could lead to vaccine hesitancy.^12-15^

The aim of this study was to investigate the knowledge and attitudes about influenza vaccination as factors to vaccine uptake and hesitancy in a population with ^18,19,20,21^ RD from the north of Mexico.

## Patients and methods

### Patients and settings

A descriptive cross-sectional study was designed. A self-administered questionnaire was applied during an educational community speech for RD patients in Monterrey, Mexico, in October 2019, where a vaccination module of information was set, and it was also applied in the rheumatology clinic of the University Hospital from the Autonomous University of Nuevo Leon in Monterrey, Mexico, between November 2019 and February 2020. We included all the patients with RD confirmed who approached and accepted to answer the questionnaire in the module from any age, who be able to read and write (regardless of educational level). We excluded in the rheumatology clinic those patients who already filled the questionnaire during the educational community speech.

The study was conducted in compliance with the Declaration of Helsinki and the Good Clinical Practice Guidelines, local ethics committee approval was waived due to the descriptive and anonymous nature of the study, permissions from the research coordination and the head of the rheumatology service were obtained. All the participants were informed of the purpose of the survey and verbal consent was obtained before their inclusion.

### Questionnaire

The questionnaire asks age (in years), RD diagnosis, and ten questions (modified from Alqahtani et al.^16^) all with two possible answers (yes/no). Questions ask about uptake of influenza vaccination, safety, efficacy, knowledge of cost-free availability, and the relationship between influenza vaccination and RD. This tool was not previously validated and the modification and translation from English to Spanish were done by the authors.

### Statistical methods

Pre-study sample size calculation was not carried out since we performed a descriptive analysis of a convenience sample. Results are presented as frequencies and percentages for categorical variables and according to distribution as means and standard deviation (SD) or median and interquartile range (IQR). The Kolmogorov-Smirnov test was performed to determine normality. The Chi-square and Mann-Whitney U tests were performed to compare groups: community vs. clinic and been vaccinated vs. never been vaccinated. A *P*-value ≤0.05 was considered statistically significant. Data were managed in Microsoft Excel and analyses were performed using IBM SPSS Statistics for Windows, version 22 (IBM Corp., Armonk, N.Y., USA).

## Results

### General characteristics

A total of 223 questionnaires were filled. One-hundred and twenty-two (54.7%) in the community setting and one-hundred and one (45.3%) in the clinic. There were missing data for age and diagnosis in 27 (22.1%) of 122 patients of the community. The total median age was 51 (IQR = 39–61.75) years. A total of one-hundred and twenty-seven (64.8%) patients had RA, twenty-one (10.7%) patients had osteoarthritis (OA), fifteen (7.7%) had systemic lupus erythematosus (SLE), twenty-three (11.7%) had other autoimmune diseases (AID) and ten (5.1%) had other non-AID, Table 1. The influenza vaccine uptake by diagnosis was 80.3% in RA, 76.2% in OA, 86.7% in SLE, 73.9% in other AID, and 60% in other non-AID. We also analyzed the characteristics between those who had been vaccinated and those who had never been vaccinated, the results were not statistically significant (supplementary material, Table S1).

**Table 1.** General characteristics

	Total, *N* = 196	Community, *N* = 95	Clinic, *N* = 101	*P*
Age, years, median (IQR)	51 (39–61.75)	55 (46–62)	45 (30–59)	0.001
18–34 years, *n* (%)	40 (20.4)	10 (10.5)	30 (29.7)	0.001
35–44 years, *n* (%)	38 (19.4)	15 (15.8)	23 (22.8)	0.216
45–54 years, *n* (%)	37 (18.9)	20 (21.1)	17 (16.8)	0.443
55–64 years, *n* (%)	45 (23.0)	31 (32.6)	14 (13.9)	0.002
≥65 years, *n* (%)	36 (18.4)	19 (20.0)	17 (16.8)	0.564
Diagnosis, *n* (%)				
Rheumatoid arthritis	127 (64.8)	67 (70.5)	60 (59.4)	0.104
Osteoarthritis	21 (10.7)	14 (14.7)	7 (6.9)	0.078
Systemic lupus erythematosus	15 (7.7)	3 (3.2)	12 (11.9)	0.022
Other AID*	23 (11.7)	8 (8.4)	15 (14.8)	0.164
Other non-AID**	10 (5.1)	3 (3.1)	7 (6.9)	0.226

IQR, interquartile range; AID, autoimmune disease.

*Including 5 Undifferentiated arthritis, 5 Rhupus syndrome, 2 adult-onset Still disease, 2 ankylosing spondylitis, 1 juvenile idiopathic arthritis, 1 dermatomyositis, 1 mixed connective tissue disease, 1 Raynaud’s syndrome, 1 primary antiphospholipid syndrome, 1 Sjögren syndrome, 1 systemic sclerosis, 1 anti-synthetase syndrome, and 1 ANCA-associated vasculitis.

**Including 5 fibromyalgia and 5 soft-tissue diseases.

### Knowledge and attitudes

#### Influenza vaccine uptake and cost awareness

From the total of patients, one-hundred and seventy-eight (79.8%) patients were vaccinated for influenza at least once, ninety-seven (79.5%) from the community patients, and eighty-one (80.2%) from the clinic patients. A total of 208 (93.3%) patients knew that influenza vaccine is freely provided, Table 2. Only seven (3.9%) patients who had been previously vaccinated did not know that influenza vaccine is freely provided vs. eight (17.8%) patients who had never been vaccinated (*P* = .001), Table 3.

**Table 2.** Questions and answers about influenza vaccination in rheumatic patients

Question	Answer	Total, *N* = 223	Community, *N* = 122	Clinic, *N* = 101	*P*
Influenza vaccine uptake and cost awareness					
Q2. Have you ever been vaccinated for influenza? *n* (%)	Yes	178 (79.8)	97 (79.5)	81 (80.2)	0.898
	No	45 (20.2)	25 (20.5)	20 (19.8)	
Q7. Do you know that the influenza vaccine is freely provided? *n* (%)	Yes	208 (93.3)	115 (94.3)	93 (92.1)	0.517
	No	15 (6.7)	7 (5.7)	8 (7.9)	
Safety and efficacy of influenza vaccination					
Q3. Influenza vaccine is safe and effective: *n* (%)	Yes	187 (83.9)	107 (87.7)	80 (79.2)	0.086
	No	36 (16.1)	15 (12.3)	21 (20.8)	
Q4. The best way to avoid complications of influenza is by using the influenza vaccine: *n* (%)	Yes	191 (85.7)	104 (85.2)	87 (86.1)	0.850
	No	32 (14.3)	18 (14.8)	14 (13.9)	
Q5. It is safe to be vaccinated for influenza and other vaccines at the same time: *n* (%)	Yes	136 (61.0)	73 (59.8)	63 (62.4)	0.699
	No	87 (39.0)	49 (40.2)	38 (37.6)	
Q6. Influenza vaccine weakens the immune system and makes me susceptible to other infections: *n* (%)	Yes	79 (35.4)	29 (23.8)	50 (49.5)	<0.001
	No	144 (64.6)	93 (76.2)	51 (50.5)	
Q8. Herbal medications, traditional medicine, and some food (like orange) are better than the influenza vaccine: *n* (%)	Yes	69 (30.9)	28 (23.0)	41 (40.6)	0.005
	No	154 (69.1)	94 (77.0)	60 (59.4)	
Q9. Influenza vaccine instead of helping me will get me worse: *n* (%)	Yes	54 (24.2)	21 (17.2)	33 (32.7)	0.007
	No	169 (75.8)	101 (82.8)	68 (67.3)	
Influenza vaccination and its relationship with rheumatic diseases					
Q1. Can rheumatic diseases patients be vaccinated? *n* (%)	Yes	206 (92.4)	114 (93.4)	92 (91.1)	0.510
	No	17 (7.6)	8 (6.6)	9 (8.9)	
Q10. Influenza vaccine will worse my rheumatic disease: *n* (%)	Yes	26 (11.7)	11 (9.0)	15 (14.9)	0.177
	No	197 (88.3)	111 (91.0)	86 (85.1)

**Table 3.** Previous vaccination, cost awareness, and relationship with rheumatic diseases

Question	Answer	Previous vaccination		*P*
		Yes (N = 178)	No (N = 45)	
Q7. Do you know that the influenza vaccine is freely provided? n (%)	Yes	171 (96.1)	37 (82.2)	0.001
	No	7 (3.9)	8 (17.8)	
Q1. Can rheumatic diseases patients be vaccinated? n (%)	Yes	164 (92.1)	42 (93.3)	0.787
	No	14 (7.9)	3 (6.7)	
Q10. Influenza vaccine will worse my rheumatic disease: n (%)	Yes	17 (9.6)	9 (20.0)	0.051
	No	161 (90.4)	36 (80.0)	

#### Safety and efficacy of influenza vaccine

A total of 187 (83.9%) patients considered influenza vaccine safe and effective. One-hundred and ninety-one (85.7%) patients thought that the best way to avoid complications of influenza is by using the vaccine. Eighty-seven (39.0%) patients answered that it is not safe to be vaccinated for influenza and other vaccines at the same time. Seventy-nine (35.4%) patients thought that influenza vaccine weakens the immune system and makes them susceptible to other infections. Sixty-nine (30.9%) patients considered other products (herbal medications, traditional medicine, or some food) better than influenza vaccine. Fifty-four (24.2%) patients thought that influenza vaccine instead of helping them will get them worse, Table 2.

From all the patients, forty-five (20.2%) had never been vaccinated for influenza, in this group, there were more concerns about safety and efficacy. From those who had never been vaccinated, twelve (26.7%) patients did not consider influenza vaccine safe and effective vs. twenty-four (13.5%) patients who had been vaccinated previously (*P* = .032). Also, eleven (24.4%) patients who had never been vaccinated did not consider influenza vaccine as the best way to avoid complications of influenza vs. twenty-one (11.8%) patients who had been vaccinated (*P* = .031). There were other differences being not statistically significant that are shown in Table 4.

**Table 4.** Influenza vaccine uptake and safety and efficacy concerns

Question	Answer	Previous vaccination		*P*
		Yes (*N* = 178)	No (*N* = 45)	
Q3. Influenza vaccine is safe and effective: *n* (%)	Yes	154 (86.5)	33 (73.3)	0.032
	No	24 (13.5)	12 (26.7)	
Q4. The best way to avoid complications of influenza is by using the influenza vaccine: *n* (%)	Yes	157 (88.2)	34 (75.6)	0.031
	No	21 (11.8)	11 (24.4)	
Q5. It is safe to be vaccinated for influenza and other vaccines at the same time: *n* (%)	Yes	114 (64.0)	22 (48.9)	0.063
	No	64 (36.0)	23 (51.1)	
Q6. Influenza vaccine weakens the immune system and makes me susceptible to other infections: *n* (%)	Yes	60 (33.7)	19 (42.2)	0.286
	No	118 (66.3)	26 (57.8)	
Q8. Herbal medications, traditional medicine, and some food (like orange) are better than the influenza vaccine: *n* (%)	Yes	55 (30.9)	14 (31.1)	0.978
	No	123 (69.1)	31 (68.9)	
Q9. Influenza vaccine instead of helping me will get me worse: *n* (%)	Yes	39 (21.9)	15 (33.3)	0.110
	No	139 (78.1)	30 (66.7)	

#### Influenza vaccination and its relationship with rheumatic diseases

Only 17 (7.6%) patients considered RD patients could not be vaccinated. Twenty-six (11.7%) thought that influenza vaccine would worsen their RD. Differences between each setting are shown in Table 2. Among patients who had never been vaccinated were more frequent the thought that influenza vaccine would worsen their RD, nine (20.0%) patients vs. seventeen (9.6%) (*P* = .051). Table 3.

## Discussion

Vaccination against preventable illnesses (such as influenza) is recommended for patients with RD by most national and international medical societies.^9,17-22^ However, vaccine uptake among these patients is low.^12-15,23-30^

In our study, 79.8% of patients with RD (including AID and other non-AID) were vaccinated for influenza at least once, this is above other studies who had evaluated influenza vaccine uptake at any time in unselected RD population, who reported as low as 0.4% in China,^30^ 28% in France,^24^ 34% in Ireland during the previous 12 months,^13^ 38% among autoimmune inflammatory joint disease patients under biologic therapy also in France,^31^ and 39.3% in a previous pilot study in our center.^28^ Taking account only the RA patients, the vaccine uptake persists high in 80.3% compared with other RA populations, who had reported an influenza vaccine uptake in the United Kingdom of 46% of patients with <65 years and 81% of patients with >65 years by Fahy et al.^23^ 57% in the previous 12 months also in the United Kingdom by Bridges et al.^12^ 59.7% in France by Hua et al.^14^ 60.1% in the international COMORA cohort being the lowest in Egypt with 28.7% and the highest in the United States with 89.2% reported by Hmamouchi et al.^26^ and 65.3% in Germany by Feuchtenberger et al.^25^

Most of the patients knew that influenza vaccine is freely provided (in Mexico), but among those who had never been vaccinated the proportion of patients who did not know it was higher, to our knowledge only the study by Jiang et al. explore the cost as a factor in RD patients, they report 3% of patients who had never been vaccinated because the vaccine was too expensive.^30^

We also found in the group of patients who had never been vaccinated for influenza more concerns about its efficacy being part of the factors that affect vaccine hesitancy; 26.7% of patients in this group did not consider influenza vaccine safe and effective, 24.4% of patients of this same group did not consider the vaccine as the best way to avoid complications, it is a high proportion compared to other reports ranging from 5% to 27%.^12-14,24,27,30,31^ In the same line of efficacy, surprisingly 30.9% of the total patients considered other options (herbal or traditional medicine and some food) better than influenza vaccine, this could be a consequence of tradition and cultural influence of prehispanic medicine in Mexico. Talking about safety concerns, another factor of vaccine hesitancy, we found that 51.1% of patients who had never been vaccinated considered not safe to be vaccinated for influenza with other vaccines at the same time, in this same group 42.2% thought that influenza vaccine weakens the immune system and makes them susceptible to other infections, and 33.3% thought that instead of helping them will get them worse. Other studies also have reported concerns about side effects of influenza vaccine, Bridges et al. reported 19% of concerns over side effects,^12^ Lanternier et al. reported 35% of fear of side effects among the principal reasons of no vaccination,^24^ Haroon et al. reported 1.8% of fear of vaccination,^13^ Hua et al. reported 39.8% (in RA) and 21% (in spondylarthritis) of fear of side effects in unvaccinated patients,^14^ Brocq et al. reported 40% of concern about adverse effects among not vaccinated patients,^27^ Harrison et al. reported 35.5% of fear of side effects in patients who declined influenza vaccination,^15^ Murray et al. reported 25% of fear of side effects.^29^ All these results should lead us to clarify and educate our patients about the real probability of mild and severe side effects of influenza vaccination.

At last, we also found that 20% of patients who had never been vaccinated for influenza thought that their RD will worsen, this factor has been reported by Harrison et al. at the same proportion 20.1%,^15^ and to our knowledge this has not been explored in other studies.

Some of our limitations are that we included RD patients in two settings, those who were invited in the community speech, which could results in a selection bias because probably only the patients who are interested in vaccination approach to the module of information, and we also invited consecutive patients who attended to their appointment in our rheumatology clinic. However, there was no difference in the vaccine uptake among these two groups. We also consider the lack of educational level documentation as a limitation in our study as a potential factor that affects knowledge and attitudes toward vaccination. Another limitation is the self-administered nature of the questionnaire and the missing data in age and diagnosis of 22.1% of the patients recruited at the community setting, we neither documented the percentage of acceptance to answer the questionnaire. One more limitation of the questionnaire is the lack of validation, we consider that this could be performed in other studies. We neither do a distinction between the treatments, this could be a factor at the time of vaccination prescription. We included patients from a single center and who attend to a rheumatology clinic, our population could be different from those who attended to primary care or internal medicine clinic.

Among our strengths are that we included AID and non-AID, but it is true that most of our patients have RA. We also evaluated the main components of vaccine hesitancy according to the WHO in our questionnaire. To our knowledge, this is the first study of its kind in our country, which gives us a real-world image of what is happening in the rheumatology clinical practice.

In conclusion, we demonstrated that concerns about safety, efficacy, and side effects are bigger among RD patients who have never been vaccinated than those who accepted vaccination. We also demonstrated that the fear of the vaccine, since it could worsen their RD, and the knowledge of the cost are important factors that diminished the vaccine uptake. All these factors related to confidence, complacency, and convenience are components of vaccine hesitancy that affects influenza vaccination uptake in RD patients. A better understanding of all these factors should lead us to develop multidisciplinary strategies to overtake these obstacles in this population and diminished their infection risk of influenza and all other vaccine-preventable diseases.
